# Balancing Data Quantity and Quality: Evaluating Curation Strategies for Bioactivity Prediction in Lead Optimization

**DOI:** 10.1021/acs.jcim.6c01018

**Published:** 2026-06-23

**Authors:** Carl C. G. Schiebroek, Gregory A. Landrum, Sereina Riniker

**Affiliations:** Department of Chemistry and Applied Biosciences, 27219 ETH Zurich, Vladimir-Prelog-Weg 2, 8093 Zurich, Switzerland

## Abstract

Building good machine-learning (ML) models to predict the bioactivity of novel chemical matter remains a challenging task. Accurate models require a training set with a large number of diverse compounds and a low level of noise. When extracting data from public databases such as ChEMBL, different levels of curation rigor may be applied, resulting in training sets of varying size, diversity, and, presumably, noise levels. It is not possible to know *a priori* whether increasing the size of the data set at the cost of adding more noise improves model generalization. To assess this trade-off, we compare three data curation and modeling approaches: (1) models trained on data for a single target, (2) models trained on target-specific data further restricted to a single set of assay conditions, and (3) multitask learning (MTL) models where each assay condition is treated as a separate task. This MTL approach was designed to bridge the gap between data quantity and quality. Graph neural networks (GNN) and random forests (RF) regressors are evaluated via a leave-assay-out strategy to minimize noise in the test sets. Our results show no meaningful performance differences between these curation strategies, suggesting that for lead-optimization tasks, increasing data quantity at the expense of label consistency does not improve generalization. Notably, the MTL approach also failed to provide a performance advantage. Additionally, we find that GNNs exhibit high seed-dependent variability in connection with the comparatively small training sets common for bioactivity measurements, highlighting the necessity of multiseed evaluation for robust model assessment.

## Graphical abstract

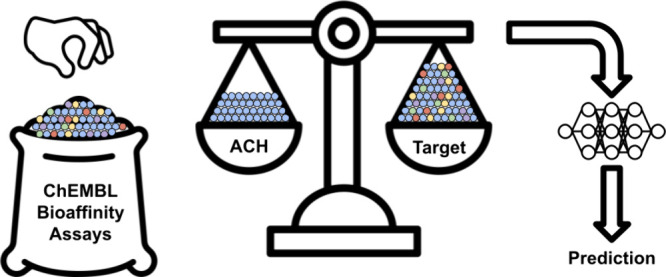

## Introduction

Predicting the bioactivity of novel chemical matter *in silico* remains a challenge in drug discovery. The development of accurate machine-learning (ML) models requires training sets containing high-quality, diverse samples. Even under tightly controlled conditions, such as a single assay protocol performed in the same laboratory, bioactivity measurements are inherently subject to experimental variability. This intralab experimental variability has been estimated to be approximately 0.3 log units (or a 2-fold difference in activity).^1–3^ However, when combining data from assays using inconsistent protocols, the level of noise is likely (much) higher.^4^ Large data sets measured in the same laboratory under the same conditions are typically only available in proprietary databases of pharmaceutical companies. In contrast, public databases such as ChEMBL^5,6^ contain collections of smaller assays performed under various experimental conditions, resulting in considerable heterogeneity in the bioactivity values recorded. This heterogeneity poses a challenge to the development of robust ML models with publicly available data.

Previous research^2–4^ has focused on quantifying the amount of experimental noise in the ChEMBL database. One can do this by taking compounds for which bioactivity has been measured for the same target in different assays, and comparing the reported values to each other. Using a minimally curated data set, Landrum and Riniker^4^ observed median absolute deviation values of 0.50 log units when comparing IC_50_ assays and 0.52 log units for K_*i*_ assays. Conversely, when a more stringent curation scheme based on identical assay metadata (consistent conditions) and additional criteria was applied, the median absolute deviation was reduced to 0.27 log units for IC_50_ data and 0.45 log units for K_*i*_ data. This demonstrates that considerable improvements in data consistency can be achieved. However, the more stringent curation strategy substantially reduces the number of available data points.

These data curation concerns are particularly relevant given the increasing use of artificial neural networks (ANNs) for molecular property prediction. Graph neural networks (GNNs), a subclass of ANNs, are commonly used for molecular property prediction because molecules can be naturally represented as graphs with atoms as nodes and bonds as edges.^7–9^ For complex tasks such as bioactivity prediction, it is assumed that large and deep networks (i.e., with many parameters) are better in capturing the nonlinear relationships between molecular structure and bioactivity. As a result, training such models typically requires the availability of large data sets to effectively fit their parameters without (too much) overfitting. Consequently, a strong emphasis is placed on data quantity when assembling training sets for these types of models. This highlights a key trade-off in training ML models: large data sets are needed to robustly fit complex models, but increasing data set size by aggregating measurements from different assays reduces overall data quality.^4^ The presence of experimental noise imposes an upper bound on the accuracy of any predictive model,^1^ i.e., the accuracy of a model cannot exceed the irreducible error in its training labels. Furthermore, evaluating models on test sets containing poor quality or heterogeneous measurements may not provide a reliable estimate of real-world performance.^10^ Therefore, special care should be taken to use high-quality, well-curated test sets to evaluate models.

This study aims to investigate the data quantity–quality trade-off by examining how different curation strategies affect ML model performance in predictions for novel chemical matter. We investigate three modeling strategies: (1) models trained on data from consistent assay conditions, (2) models trained on data aggregated across multiple assay conditions, and (3) a multitask learning (MTL) approach where each assay condition is treated as a separate task. The MTL framework is intended to leverage the larger volume of the target aggregated data while maintaining the lower variability of assays with consistent conditions, potentially balancing data quantity with data quality. The topic of data selection for ML has been studied before, e.g., in the context of training “local” and “global” ADME models.^11^ Local models are trained on a small set of similar compounds, whereas global models are trained on a larger set of diverse compounds. However, in this case, all labels are obtained from the same assay, and therefore the same level of noise is expected between the training sets for local and global models. Combining data from different sources by distinguishing them as different tasks has also been explored in the space of ADME prediction using industry project data sets.^12^ These differ from bioactivity data with different assay conditions found in ChEMBL in a few key ways. First, the number of data points per task is much larger for ADME project data in industry, compared to IC_50_ or K_*i*_ data for a specific target with different assay conditions recorded in ChEMBL. Second, given that ADME properties are measured for compounds from different projects, the chemical diversity of these sets is expected to be considerably larger. Another approach utilizing data from different sources that has proven effective for ADME predictions is pretraining on proprietary data, followed by fine-tuning on the data set of interest.^13^ In this case, the pretraining set is internally consistent, allowing the model to learn from a larger number of samples without necessarily introducing additional noise, provided an appropriate fine-tuning approach is employed. In our experiments, we implemented a leave-assay-out strategy to ensure minimal noise in our test sets. This means that models were trained on all curated assays for a specific target and measurement type (IC_50_ or K_*i*_), leaving one assay out for testing. As each test set is comprised entirely of compounds from a single assay, the variability within the test set is minimized, ensuring high-quality test sets. Through a careful evaluation of data curation strategies using commonly used regression algorithms, we aim to enable users of ChEMBL bioactivity data to make more informed decisions when building ML models for lead optimization.

## Methods

### Extracting Data from ChEMBL34

The data extraction protocol was adapted from ref 4. Briefly, a local copy of the PostgreSQL dump of ChEMBL, version 34^14^ provided by the ChEMBL team was used without modifications. Standard SQL queries, in combination with Python code were used to construct the data sets. The code to construct the extracted data sets is provided on GitHub: https://github.com/rinikerlab/ChEMBL_MTL.

### Data Curation Schemes

For this study, two data curation schemes were used. The following general curation criteria were applied:

- Only K_*i*_ **or** IC_50_ data
- One selected target (ChEMBL ID)
- Only include data with confidence level = 9
- Only data with unit in nM, no data validity comment, no relative data (only “=” accepted), for a single protein
- No mixing of mutant and wild-type data in one data set
- Only one assay per document per data set
- Molecules are standardized (using RDKit’s^15^ ChargeParent standardization)
- Pairs of entries for the same molecule with an exact difference in pChEMBL value of 3, 6, or 9 were removed (likely copies with a unit mistake made by the copier)
- For pairs of entries for the same molecule in the same assay, the one with the highest pChEMBL value was kept
- For pairs of entries of the same molecule over different assays, the one from the assay with the lowest ChEMBL ID (proxy for oldest) was kept

The curation scheme following just these steps (i.e., curating data for a specific target) is referred to as “target curation” in this work. In the second curation scheme, termed “assay conditions hash (ACH) curation”, an additional criterion for consistent assay conditions was set. The ACH is explained in detail in ref 4. In short, the assay metadata were hashed, and only assays with the same assay metadata (and thus, same ACH) were combined. This assay condition hash and its encoded metadata are visualized in Figure 1A.

**Figure 1:**
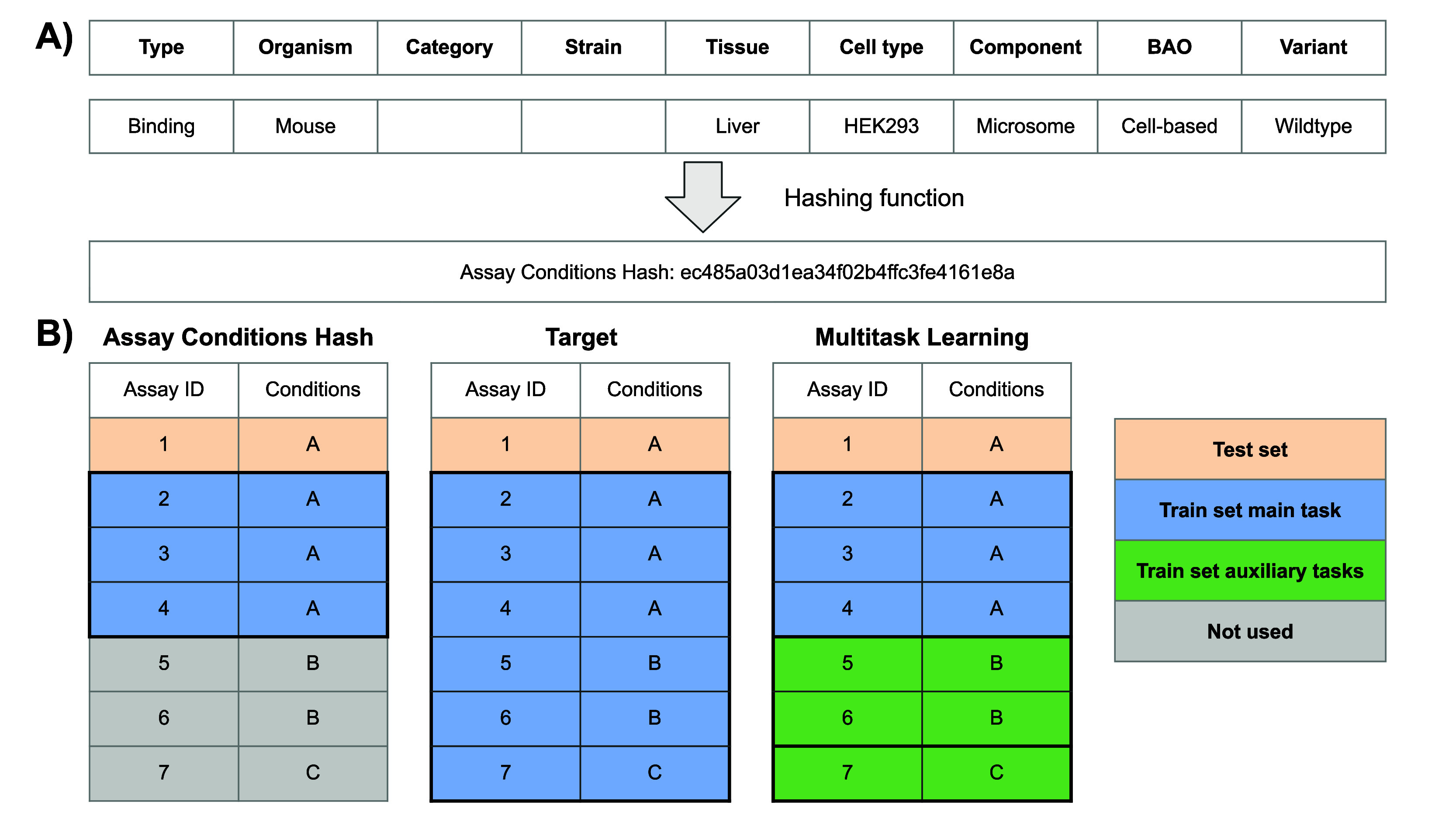
(A) Hashing of assay metadata into the assay conditions hash (ACH). (B) Schematic visualization of the curation strategies ACH (left), target (middle), and MTL (right) and the corresponding data splitting. All models are evaluated on the same test set, i.e., in the example assay 1 with conditions A. For ACH, a model is trained on assays 2–4 with the same assay conditions as the test set (conditions A). For target curation, a model is trained on assays 2–7 with mixed assay conditions (conditions A–C). For MTL, a model is trained for three tasks simultaneously (tasks A, B, and C), representing prediction of bioactivity under different assay conditions. Only predictions for the task with conditions corresponding to the test set (condition A) are evaluated on the test set.

The following metadata were considered for the ACH:

- Assay type (binding/functional)
- Cell type (e.g., HEK293)
- Assay subcellular fraction (e.g., cell membrane)
- BAO format (e.g., single protein format)
- Assay tissue (e.g., blood)
- Assay organism (e.g., *Rattus norvegicus*)
- Assay strain (e.g., Sprague–Dawley)
- Assay category (e.g., confirmatory)
- Variant ID

Note that not all metadata is always available in ChEMBL. While assay type and BAO format are always provided, other fields are often left empty. In these cases, only assays that match in the provided metadata and miss the same metadata fields were grouped together. By including the variant ID in the ACH and removing all entries that contain words like “mutant” or “variant” in the description and do not have a variant ID, we try to ensure that the mutant/variant type within one ACH remains consistent. For the target curation scheme, all entries that contain words like ‘mutant” or “variant” in the description were removed, regardless of any potentially assigned variant ID.

The two different curation strategies allow two types of models to be trained: (i) a ‘target” model trained on data obtained using target curation, and (ii) an ACH model trained on data obtained using ACH curation. Additionally, a third setup was explored using MTL, wherein each distinct ACH was considered a task.

### Splitting the Data Sets

Curated subsets were obtained from ChEMBL34 ^14^ using the aforementioned curation criteria. For each target, we used individual assays as test sets in order to remove any noise caused by interassay variability from the test data. Test-set assays were chosen following a few criteria. First, the assay must have more-than-minimal metadata information, meaning at least one optional metadata column must be specified (most often the cell line). This ensures that the ACH contains useful information and does not simply group assays together where no metadata is specified. Additionally, an assay must contain 20–50 compounds to be useful as a test set. Fewer compounds would not produce stable performance metrics, while more compounds likely do not originate from a single medicinal chemistry paper with primary data. If there was overlap between the compounds in the test assay and the training assays, the overlapping compounds were removed from the training set. Finally, data sets were only used for training the ML models if, after splitting the test set and the validation set (see the next paragraph), there were still at least 500 data points for training. This resulted in 422 assays, of which 97 had IC_50_ data and 325 had K_*i*_ data. These assays spanned 24 different targets, with 6–46 test assays per target (median of 16 test assays per target).

The data splitting and model set up is visualized in Figure 1B. The validation set used in the model training was selected using a clustering scheme adapted from ref 16. Briefly, Morgan bit fingerprints with a radius of 3 were generated using RDKit (2023.09.5).^15^ The Butina algorithm^17^ (as implemented in RDKit) was then used to cluster the data with a Tanimoto distance threshold of 0.65. Small clusters (less than five compounds) were merged with the nearest bigger cluster. The clusters were sorted by size, and compounds were picked by iterating over the clusters (in the order of decreasing size) until the desired amount of compounds was reached (here 10% of the total training set). The validation set was only used for early stopping when training the GNNs. For training the RFs, no validation set was split from the training data.

### Training the Models

#### Graph Neural Networks (GNNs)

The AttentiveFP architecture^8^ as implemented in PyTorch Geometric^18^ (version 2.5.0) was used with the following model parameters: 200 hidden channels, 5 layers, time step of 2, dropout of 0.3 (unless explicitly stated otherwise). Graphs were featurized as described in ref 19. Models were trained using an Adam optimizer^20^ (as implemented in PyTorch v2.2 ^21^) with an initial learning rate of 1e-3 and weight decay of 1e-5. The ReduceLROnPlateau from PyTorch was used with a reduction factor of 0.99 and patience of 2 epochs. A batch size of 32 was employed. The effect of hyperparameter optimization on the predictive performance of the validation set was investigated for a representative target (selected based on the training set size being close to the median of all training sets). No meaningful benefit of hyperparameter tuning was observed, and therefore the selected hyperparameters were kept and no further tuning was performed. Models were trained for 1000 epochs, or until the training and validation loss converged (i.e., did not change over the last 200 epochs). The best model was then picked based on the validation loss (MSE). For the MTL models, only the main task (with the same ACH as the test set) was considered for the early stopping criterion. Each model was trained and evaluated using five different random seeds for initialization of the weights.

#### Random Forest (RF) Regressors

The RandomForestRegressor implementation of sklearn (v1.4)^22^ was used, with “sqrt” max features, 300 estimators, and no limitation on the maximum depth. Models were evaluated on the same test sets as the GNNs, and trained on the combined training and validation set as used for the GNNs. Morgan count fingerprints with a radius of 2 and a size of 2048 bits were used as descriptor.

### Analysis: Spatial Statistics

To characterize the data sets obtained using the target and ACH curation schemes, *∑G* and *∑F*′ values were calculated for the different data sets, as described by Landrum et al.,^16^ and first applied in chemistry by Rohrer and Baumann.^23^ Details are provided in the aforementioned publications. In brief, G­(t) quantifies test–test distances, while F′(t) quantifies train-test distances, based on cumulative distribution functions (CDFs) of the underlying similarity distributions. Higher values of *∑G* indicate a more “clumped” test set with high overall similarity, while lower values indicate large diversity within the set. Higher values of *∑F*′ indicate higher train-test similarity. In ref 4, it was found that different types of data splitting (temporal, random, and neighbor) exhibited different profiles of *∑G* and *∑F*′. Random splits give comparatively higher *∑F*′ values, while temporal and neighbor splits give comparatively higher *∑G* values, with temporal splits being generally characterized by higher *∑G* and *∑F*′ values. These distributions indicate that in temporal splits, test compounds tend to be similar to each other, and more distinct from the training compounds compared to random splits.

## Results and Discussion

### Data Curation

ChEMBL data was curated according to the target and ACH schemes as explained in Methods. By finding compounds that were measured for the same target in different assays, we can estimate the amount of noise in the curated data (Figure 2).

**Figure 2:**
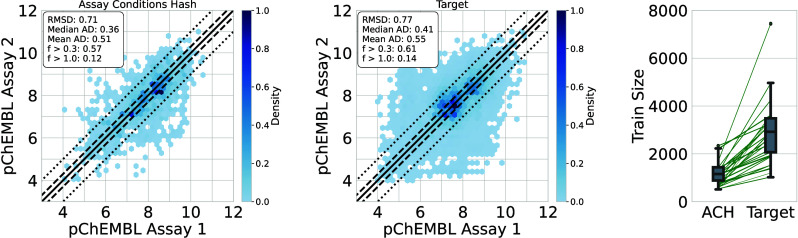
Quantity and quality of data from the two curation schemes: Density scatter plots of pChEMBL values for compounds with measurements in different assays using the ACH (left) and target (middle) curation. (right) Amount of training data obtained using the two schemes. The green lines link the amount of training data for the same data set using both curation schemes.

Using the ACH curation scheme results in somewhat better agreement between duplicate bioactivity measurements compared to the target curation scheme. The distributions of differences are significantly different from each other (Kolmogorov–Smirnov test *p*-value <0.001 ), with overall small differences for assay pairs obtained with ACH curation. This is exemplified by the lower mean (0.51 log units for ACH and 0.55 log units for target) and median (0.36 log units for ACH and 0.41 log units for Target) absolute deviation, as well as a lower fraction of deviations that are higher than the assumed experimental variability of 0.3 log units (0.57 for ACH and 0.61 for target) and the fraction of deviations that are larger than 1 log unit (0.12 for ACH and 0.14 for target). While statistically significant, the differences in these metrics between ACH and Target curation are relatively small. On the other hand, available data roughly doubles when considering the target curation criteria compared to ACH curation (right panel in Figure 2). These results characterize the data quantity-quality trade-off between the ACH and target curation scheme, and confirm that combining only assays with the same assay metadata reduces the noise in obtained data sets, as found in previous work.^4^ Note that the curation schemes here differ from the “minimal” and “maximal” curation schemes in ref 4, with less strict curation criteria for ACH compared to “maximal” curation, and more strict criteria for target compared to “minimum” curation, leading to a smaller difference between ACH and target curation than between maximal and minimal curation.

Comparing the *∑G* and *∑F*′ values from spatial statistics^23^ obtained for target and ACH curation to previous work^16^ (Figure S1 in the Supporting Information), our methods of data splitting yield spatial statistics profiles similar to those obtained with a temporal split. Temporal split validation is the gold standard for the evaluation of models in medicinal chemistry, as this best assesses real-world usefulness (prospective use).^24^ Therefore, leave-assay-out can be a reasonable approximation of this task for validating models where no temporal data is available.

Additionally, we observed similar train-test similarity (as measured by *∑F*′ based on Tanimoto similarity using Morgan bit fingerprints with radius 3) for both curation schemes, with slightly higher average train-test similarity for target curation, indicating additional data aggregation does not meaningfully increase the train-test similarity.

### Model Performance

We trained GNNs and RF regressors using the target and assay conditions hash (ACH) curation schemes and evaluated the different models on 422 different test sets. Additionally, we trained a MTL GNN, where each task was a set of assay conditions (i.e., a unique ACH). Each model was trained with five different random seeds, and performance metrics were averaged over the different seeds, and distributions over the test sets were compared. The result for the median performance metric over all test sets is shown in Figure 3 for median absolute error (medAE) and Kendall’s τ. Full distributions are shown in Figure S2 in the Supporting Information. As a baseline for the lower limit of model performance, we assumed the mean bioactivity value of the training set for each data point in the test set. As the different data curation strategies result in different training sets, the mean of the training set also slightly differs (Figure S3 in the Supporting Information), leading to slightly different performance (Figure S4). However, as the mean of the training set using target and ACH curation only differs slightly, only predicting the mean of the training set for the target scheme is shown here.

**Figure 3:**
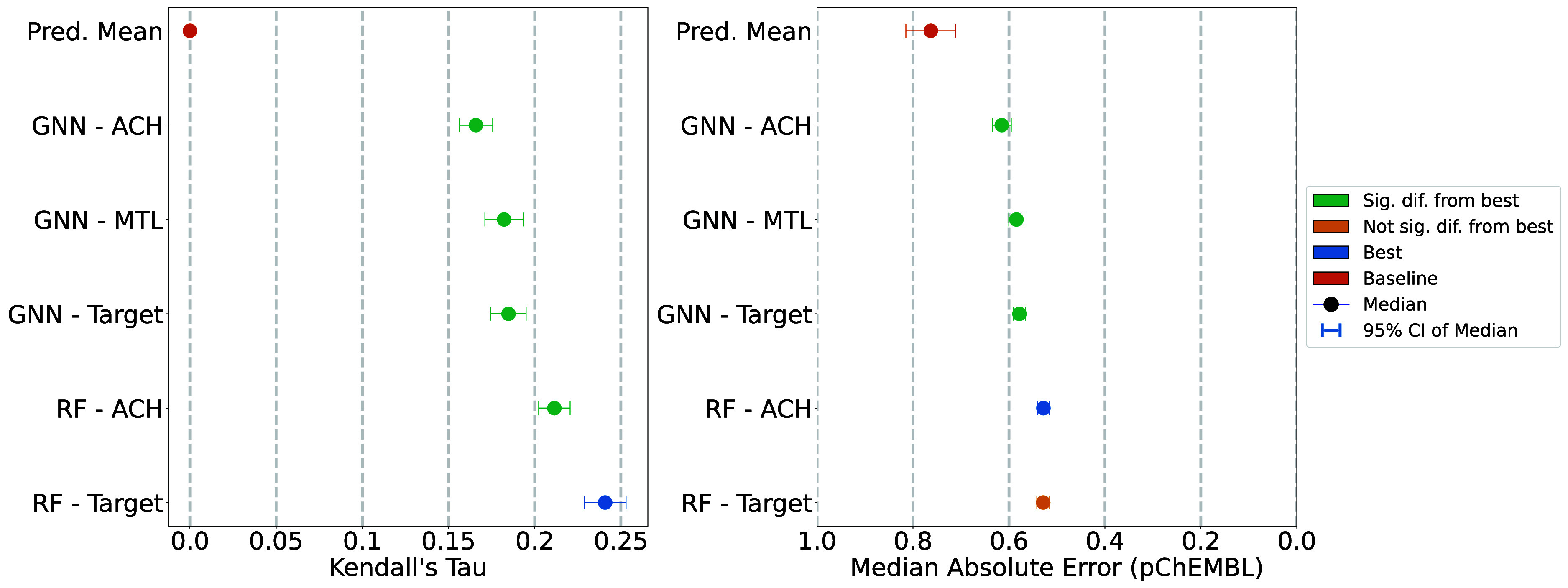
Comparison of the performance of the ML models over the 422 test sets. The best method is determined based on the best median value of the performance metric. All other methods are compared to this best method using the Conover-Friedman^25,26^ post hoc test with Bonferroni correction^27^ for multiple comparisons (α = 0.05) as implemented in scikit-posthocs.^28^ Performance is put in context of predicting the mean of the (target) training set. Left: Kendall’s τ. Right: median absolute error (medAE).

The Friedman χ^2^-test^29^ as implemented in SciPy 1.12.0 ^30^ was employed to detect if there was a statistically significant difference between any of the model pairs. The Conover-Friedman post hoc test^25,26^ with Bonferroni correction for multiple comparisons^27^ was used to compare the pairs to each other. Based on Kendall’s τ, the best performance (highest median over the 422 test sets) was observed for the RF–target model, being significantly different from all other models. For median absolute error, the RF–ACH model performed best (lowest median over the 422 test sets), with no significant (*p* < 0.05) difference to the RF–target model, while the GNN models were all significantly worse than the RF–target model. To assess if one model consistently outperforms another over all test sets, we compared the performance of each model directly against each other per test set (Figure S5 in the Supporting Information). We found no model that consistently outperformed the others over all test sets.

Overall, the models performed quite poorly in the leave-assay-out experiments. It is possible that the composition of the test set plays a role in this. Compounds within a single assay are generally highly similar to each other, with a median Tanimoto similarity (based on Morgan count fingerprints with a radius of 2) of 0.51 (Figure S6 in the Supporting Information). In contrast, compounds measured against the same target but originating from different assays showed a much lower median similarity of 0.20, close to the expected similarity for random compounds drawn from ChEMBL^31^ (Figure S6). Furthermore, the range of pChEMBL values within these test assays was relatively narrow, with a median range of 2.5 log units (95% credibility interval of the median: 2.3–2.6) (Figure S7 in the Supporting Information). Considering the assumed experimental variability of 0.3 log units within the same assay,^1^ even a theoretical model comparing repeated experimental measurements would likely yield a Kendall’s τ below 1.0. However, even in this context, all models still perform overall poorly at ranking the compounds in the test sets. Besides the test set composition, the high number of trainable parameters of the models compared to the number of training points is expected to lead to overfitting, negatively impacting the model performance.

Interestingly, the MTL approach did not yield better results than its target and ACH counterparts, despite the theoretically beneficial setup designed to maximize the number of training points while maintaining distinction between different ACH. One potential reason for this is the model architecture. The MTL framework allows for bigger training sets, which in turn can lead to better hidden representations of the molecules. However, the final prediction head, which is crucial for the correct prediction of bioactivity from the hidden representation, is still only trained on a small number of compounds with the same assay condition. Additionally, all data sets used in this work were still orders of magnitude smaller than the number of parameters in the GNNs. While the individual trees in the RF regressor models are also prone to overfitting (especially when the maximum depth of the trees is not constrained, as in this experiment), the ensemble nature of the algorithm appears more robust to overfitting in such low-data regime. These results differ from previous studies, which have shown that using MTL to combine data from different sources can improve prediction quality for ADME.^12^ However, ADME data is likely to be (much) more chemically diverse, and the number of data points per tasks was much larger than in this work. Additionally, in the experimental setup evaluated in this work, no pruning of tasks was performed, and MTL models were therefore trained on many tasks (median: 28, 95% CI of median 26–29), with most tasks contributing a small number of data points (median: 12, 95% CI of median 12–13).

### Model Consistency

To evaluate the effect of the random seed on model performance, we evaluated all models with five different random seeds. The spread was defined as the difference in performance when comparing the best (lowest medAE) to the worst (highest medAE) result over the different seeds, keeping all other factors the same (i.e., same data and model setup). By evaluating this spread for each curation strategy and model type, we assessed how sensitive different ML methods are to the random seed (Figure 4 for models obtained using ACH curation and Figure S8 in the Supporting Information for the others). Differences between spreads observed for the different curation strategies (ACH, target, MTL), model types (RF regressors, GNNs with dropout, GNNs without dropout), and data sets (training, validation, test) were compared to each other using the Friedman χ^2^-test with the Conover-Friedman posthoc analysis (α = 0.5) to evaluate significant differences between each pair.

**Figure 4:**
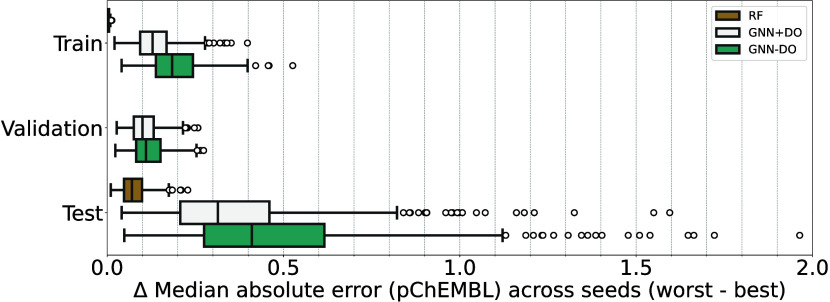
Box plots of the distributions of variability across 422 test sets using ACH curation. Variability is quantified as the difference between the maximum and minimum median absolute error (medAE) in pChEMBL obtained across five random seeds. Results are shown for the training, validation, and test sets, for RF regressors (orange), and for GNNs trained with a dropout rate of 0.3 (GNN+DO, white) and without dropout (GNN-DO, green). Note that for RF models, no validation set was used, and the training sets for these models are equal to the combined training and validation set as used for the GNNs.

We found that the different model types exhibit large differences in the variability of their performance with different random seeds. For RF–ACH models, the observed median variability was 0.07. For GNNs trained without dropout (GNN-DO), the observed median variability of 0.41 is statistically and meaningfully higher. Introducing a dropout rate of 0.3 in training significantly and meaningfully reduced the median variability to 0.31. Similar trends are observed for MTL and target models (Figure S8). The difference in variability between the GNNs and RF regressor models is likely in part due to RFs already using averaging over multiple estimators (trees). The variance between five different ensembles of 300 estimators each is expected to be smaller than the variance of five different models with a single estimator. Additionally, we observed statistically and meaningfully lower variability when evaluating the GNN–ACH models on the validation sets compared to the test sets, with median variability of 0.10. Within each model type (RF regressor or GNN), there was no statistically significant difference in variability on the test set between models trained with different curation types. Note that the validation set in these experiments was designed to be highly similar to the training set. Therefore, overfitting the training data is not expected to majorly impact predictive performance on the validation set. The test set, on the other hand, is (by design) very homogeneous and often more distinct from the training data. Therefore, overfitting the training data in slightly different ways can have a disproportionally large impact on predictions of the test set.

## Conclusions

We evaluated two curation strategies for extracting bioactivity data from ChEMBL, either aggregating all assays for a target, or only aggregating assays with matching assay metadata. Additionally, we investigated a multitask learning (MTL) strategy, where each set of assay conditions was considered a separate task. We observed a lower level of variability of bioactivity data within assays with the same ACH compared to within any pair of assays for the same target. However, aggregating only bioactivity data with the same ACH drastically decreases the amount of available data compared to aggregating over all available assays for a certain target. Evaluating both GNNs and RF regressors on bioactivity prediction using leave-assay-out with these curation strategies revealed no large differences in performance between these model types and curation strategies. Surprisingly, the MTL method did not overcome the issue of the data quality–quantity trade-off, and performed similarly to the single-task GNNs. Additionally, we observed a large random-seed-dependent variability in performance of the GNNs on the test sets. It is therefore crucial to evaluate more complex ML methods like neural networks using multiple random seeds when working with training sets of the size typically seen with bioactivity measurements. The greater variability and computational cost of GNNs relative to RF models, without a clear improvement in performance, suggest that RF models may be the preferable first choice for bioactivity prediction in lead optimization using public data.

## Supplementary Material



## Data Availability

The code used to extract the data, prepare the data sets, train and evaluate the models is available on GitHub (https://github.com/rinikerlab/ChEMBL_MTL). The data sets, both as CSVs and PyTorch graphs, as well as the performance metrics as obtained in this study are provided on the ETH Research Collection (https://doi.org/10.3929/ethz-c-000801800).

## Notes

Additional figures describing characteristics of the used data sets and more detailed model performance results (PDF)
