# Supplementary material for: Balancing Data Quantity and Quality: Evaluating Curation Strategies for Bioactivity Prediction in Lead Optimization
Source: J Chem Inf Model. 2026 Jun 23;66(13):7446–52. doi: 10.1021/acs.jcim.6c01018 (PMC13370779; doi:10.1021/acs.jcim.6c01018)
Supplement: Supplementary file 1 [file ci6c01018_si_001.pdf]

SUPPORTING INFORMATION
Balancing Data Quantity and Quality: Evaluating Curation Strategies for Bioactivity Prediction in Lead Optimization

Carl C. G. Schiebroek,[a] Gregory A. Landrum,[a], and Sereina Riniker*[a]

[a] *Department of Chemistry and Applied Biosciences, ETH Zürich, Vladimir-Prelog-Weg 2, 8093 Zürich, Switzerland. E-mail: sriniker@ethz.ch*

Contents

S1 Spatial Statistics **S2**

S2 Performance Metrics of Full Distributions **S3**

S3 Baseline Performance **S4**

S4 Performance per Test Set **S6**

S5 Intra- Versus Inter-Assay Similarity **S8**

S6 pChEMBL Range in Test Sets **S9**

S7 Model Consistency **S10**

S1 Spatial Statistics

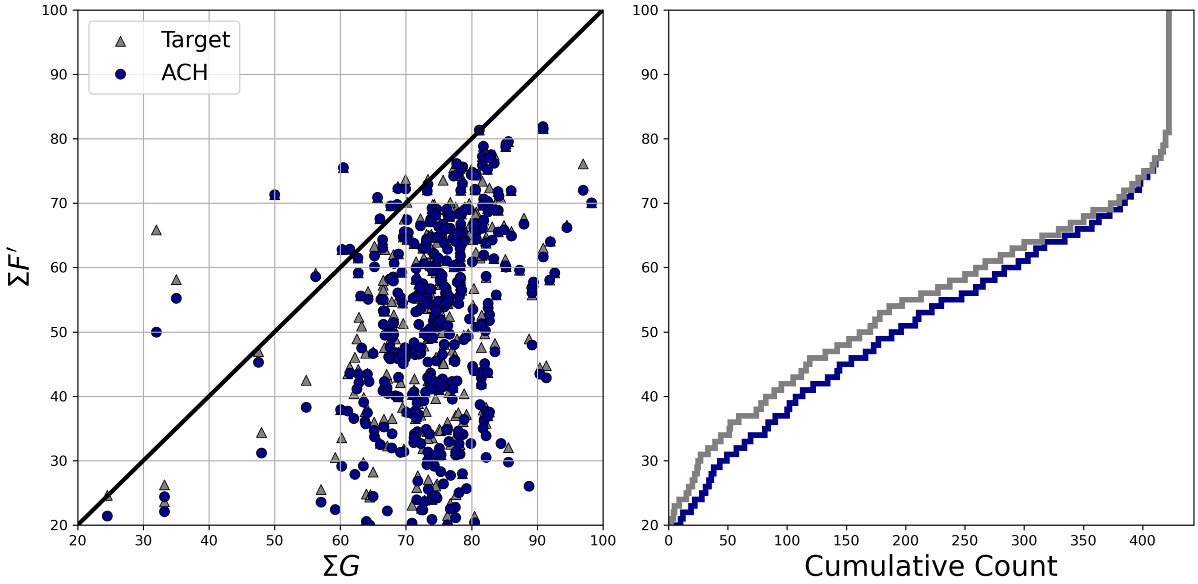

Figure S1: Spatial statistics summary plot of $\sum G$ against $\sum F'$ for the data sets used in this work for target (grey triangles) and ACH (blue circles) curation.

Datasets obtained using target and ACH curation were evaluated using $\sum G$ and $\sum F'$. These values were calculated as described by Landrum *et al.* [1]. $G(t)$, quantifying test-test distances, describes the clumpiness of the test set (i.e., higher $\sum G$ values means more clumpy test set). $F'(t)$, on the other hand, measures train-test distances, with higher values for $\sum F'$ indicating higher train-test similarity.

Landrum *et al.* [1] calculated the $\sum G$ and $\sum F'$ values for data splits obtained using different data splitting methods, namely random splits, neighbor splits, and temporal splits. For the random splits, they observed a more-or-less linear relationship between these values, with generally slightly higher $\sum F'$ than $\sum G$ values. For neighbor splits, on the other hand, they observed a similar trend, but with generally lower $\sum G$ values compared to $\sum F'$. For temporal splits, only relatively high values for $\sum G$ were observed (60-90), with varying values for $\sum F'$, and no clear relation between $\sum G$ and $\sum F'$ values.

For the leave-assay-out data splitting strategy used in this work, no clear relation between $\sum G$ and $\sum F'$ values was observed, with generally high $\sum G$ values (with a few exceptions going as low as approximately 25). As the test set for the two curation strategies is the same, we obtain the same $\sum G$ values for ACH and target curation. For $\sum F'$, slightly higher values were observed for datasets obtained using target curation (median: 55.9, 95%CI of median 54.5-57.4) compared to ACH curation (median of 52.9 95%CI of median 50.4-55.0).

These results indicate that leave-assay-out results in a splitting of the data with characteristics similar to that of a temporal split. Additionally, aggregating assays over different assay conditions for the same targets does not meaningfully increase the train-test similarity, compared to only aggregating assays with the same assay conditions.

S2 Performance Metrics of Full Distributions

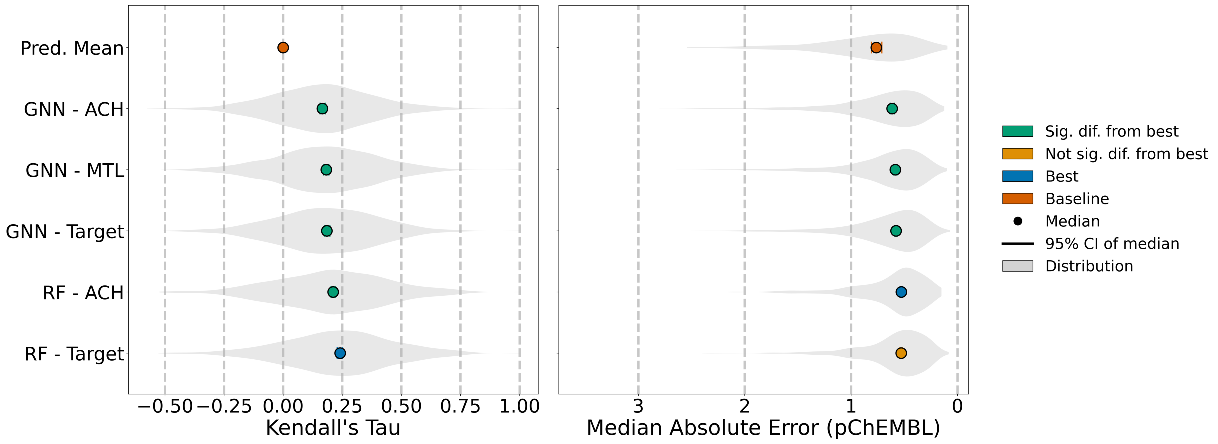

Figure S2: Comparison of the performance of different models over the 422 test sets. The best method is determined based on the best median value of the performance metric. All other methods are compared to this best method using the Conover-Friedman post hoc test[2, 3] with Bonferroni correction[4] for multiple comparisons (α = 0.05). Performance is put in context of predicting the mean of the (target) training set. Left: Kendall's τ. Right: Median absolute error (medAE). The full distributions are shown in gray.

We evaluated the different models on 422 different test sets. We trained GNNs and RF regressors using the target and ACH curation strategies. Additionally, we trained a MTL-GNN, where each task is a set of assay conditions (i.e., a unique ACH). Every model was trained with five different random seeds. Performance metrics were averaged over the different seeds and the distributions over the test sets were compared.

The distribution of the performance metrics span a wide range, from worse than random (Kendall's $\tau < 0$) to near perfect (median absolute error (medAE) < 0.3 pChEMBL), with similar distribution shapes for the different types of models. The Friedman χ^2-test[5] was employed to detect if there is a statistically significant difference between any of the model pairs. The Conover-Friedman post hoc test[2, 3] with Bonferroni correction[4] for multiple comparisons was used to compare the pairs to each other. Based on medAE, the RF-ACH model performed best (lowest median over the 422 test sets), with no significant ($p < 0.05$) difference to the RF-target model, while the GNN models were all significantly worse than the RF-target model. For Kendall's τ, the best performance (highest median over the 422 test sets) was observed for the RF-target model, being significantly different from all other models. The similar distributions for the different models can potentially be distributed to the test sets themselves, ranging in difficulty from easy to hard, depending on the relationship between the test set and available training data.

S3 Baseline Performance

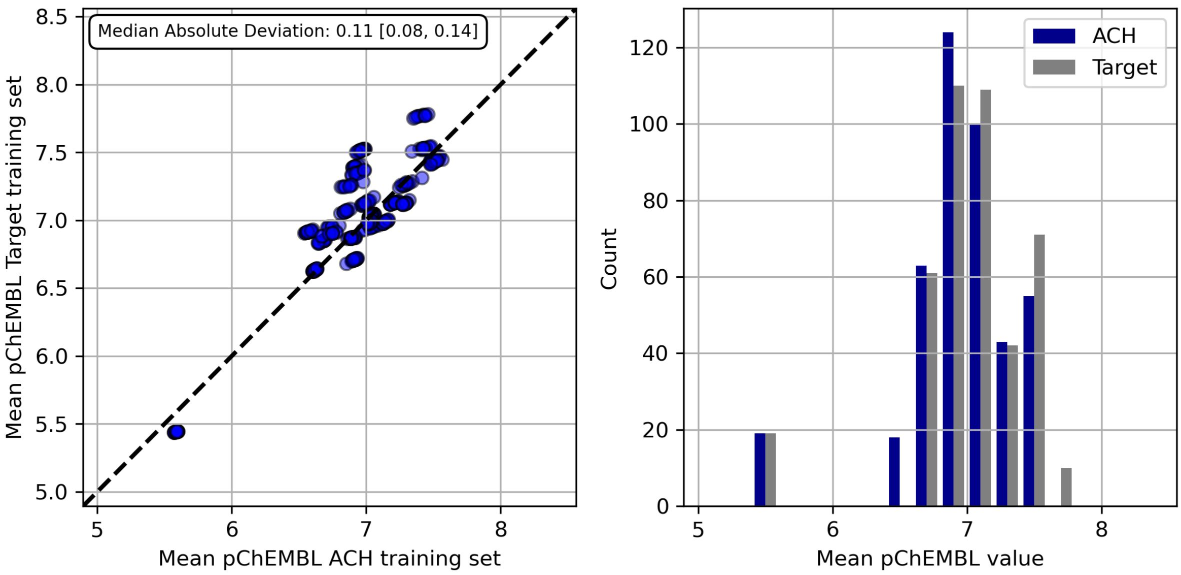

Figure S3: (Left): Comparison of the mean pChEMBL value of the training sets when obtained using ACH curation versus using target curation. (Right): Distribution of mean pChEMBL value of the training sets obtained with ACH (blue) and target (gray) curation.

To put the performance of the models in context, a minimum baseline was generated by simply predicting the mean pChEMBL value of the training set for each data point in the test set. However, as the two curation schemes give different training sets, the mean pChEMBL value can differ. The left panel in Figure S3 compares per test set the mean pChEMBL value of the corresponding training sets obtained with the two curation strategies. The median absolute deviation between the mean pChEMBL values is 0.11 (CI of median: 0.08-0.14), with generally slightly higher values for the training sets obtained using target curation. The overall distributions of the mean pChEMBL values are given in the right panel of Figure S3.

The baseline performance of using the mean pChEMBL value of the training set as prediction for the data points in the test set is show in Figure S4 for medAE (Kendall's τ is not shown because it will always be zero). The baseline medAE values with target and ACH curation are similar and significantly worse than the ML models.

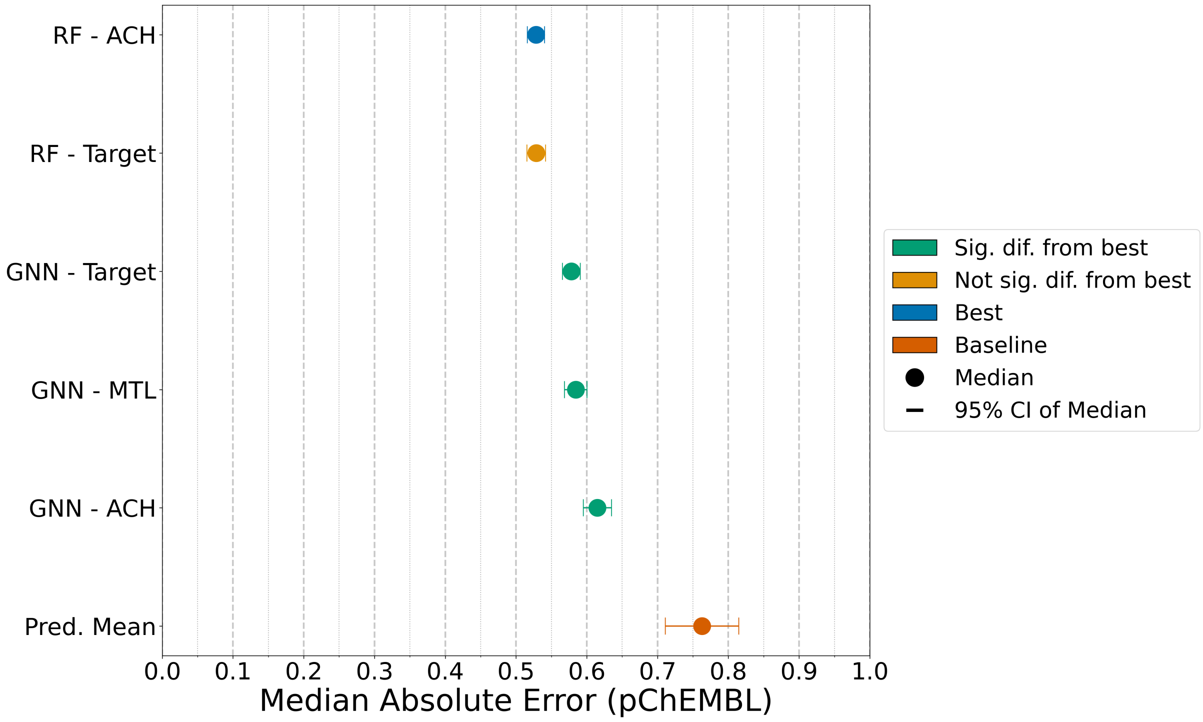

Figure S4: Comparison of the performance of the ML models and the two baseline models over the 422 test sets. The best method is determined based on the best median value of medAE. All other methods are compared to this best method using the Conover-Friedman posthoc test with Bonferroni correction for multiple comparisons ($\alpha = 0.05$).

S4 Performance per Test Set

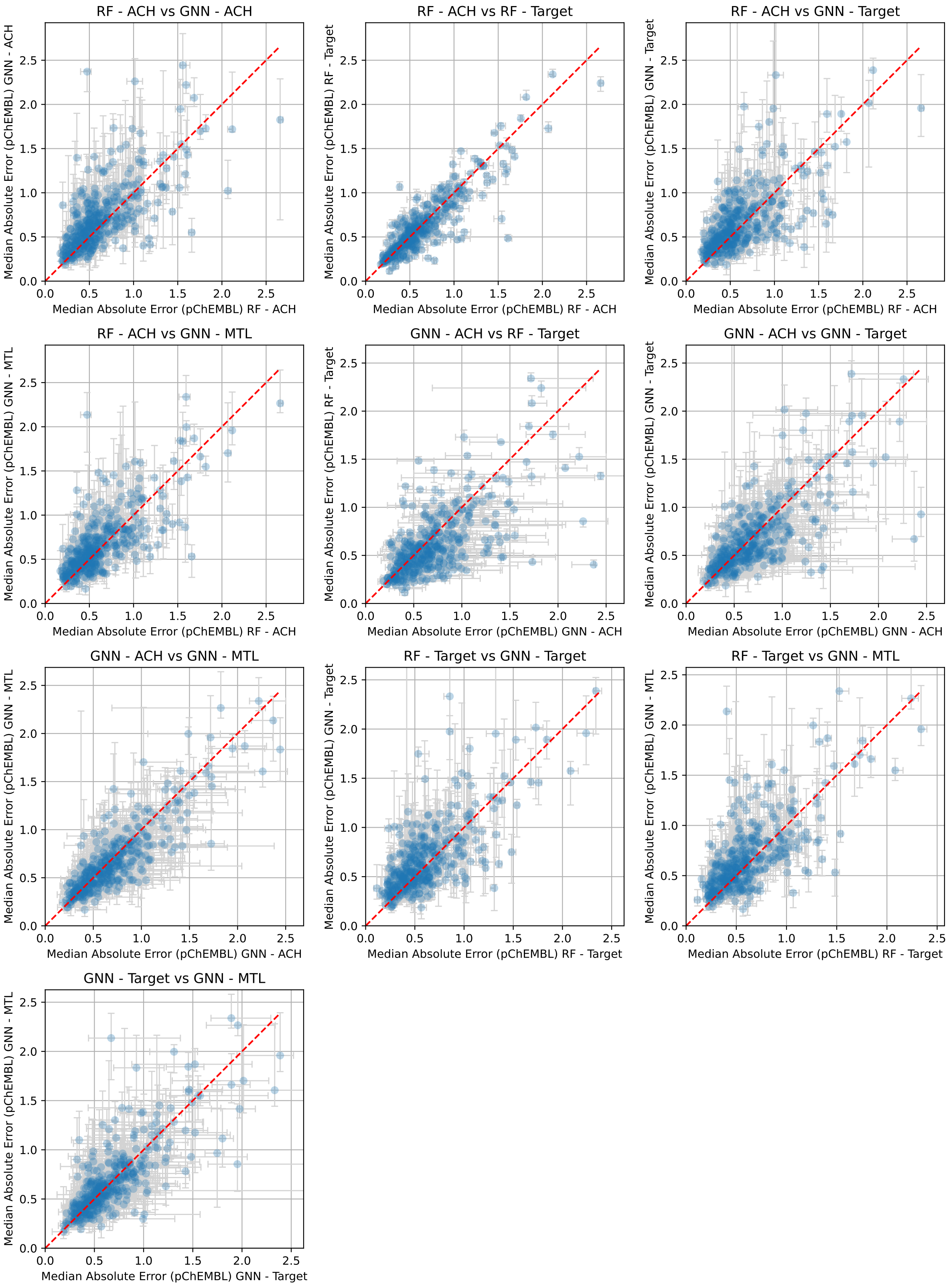

Figure S5: Performance per test set for all model pairs. Blue dots: median, gray bars: min-max value over five different seeds.

We compared the performance of each model directly against each other model per test set (Figure S5). No model achieved consistently better performance over another model over all test sets. Additionally, the large error bars for the GNNs show the large seed variability for these models. This means that, while there are trends — RF regressor models generally outperform the GNNs models slightly –, no model was consistently best. Investigation of potential underlying drivers for model performance, such as differences in training-set size and train-test similarity, detected no clear trends. Considering the relatively high computational cost and complexity of GNNs, we advise to use RF models for tasks with small training sets such as bioactivity prediction in lead optimization.

S5 Intra- Versus Inter-Assay Similarity

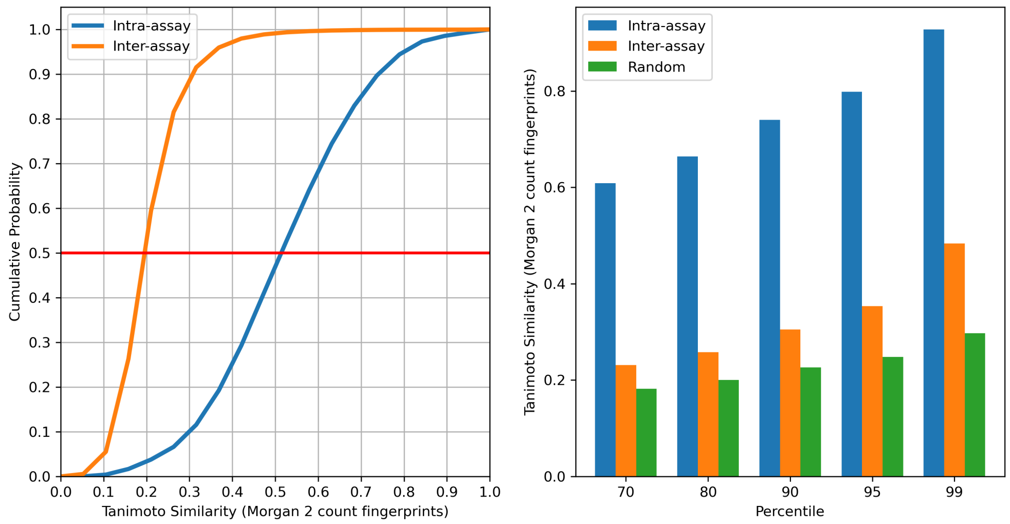

Figure S6: Distributions of intra- and inter-assay Tanimoto similarities obtained using Morgan count fingerprints with radius 2. (Left): Cumulative distribution functions (CDFs) for all within-target pairwise similarities for compounds reported in the same assay (intra-assay, blue) and in different assays (inter-assay, orange). The solid red line indicates a cumulative probability of 0.5 (median). (Right): Comparison of intra- and inter-assay similarities at the 70th, 80th, 90th, 95th, and 99th percentiles alongside expected random similarities, as described in Ref. 6.

To assess the hypothesis that compounds within one assay are more similar to each other then compounds with bioactivity measurements for the same target but originating from different assays, we calculated pairwise similarities between all compounds for each target. We then split the similarities into two categories depending on wether the compounds within the pair originated from the same assay (intra assay) or not (inter-assay). We observed a generally much higher similarity for compounds originating from the same assay (median Tanimoto similarity with Morgan count fingerprints with radius 2 = 0.51), compared to compounds from different assays (median Tanimoto similarity = 0.2) as can be seen in the left panel of Figure S6.

We also compared the values for different percentiles to those obtained from drawing random pairs of compounds as described in an RDKit blogpost [6]. As expected, the intra-assay similarity is much lower then the inter-assay similarity, as one single assay is generally extracted from a single medicinal chemistry paper, often focusing on quantitative structure-activity relationships (right panel in Figure S6). The inter-assay similarity is still slightly higher than random, most likely due to the fact that the compounds are still related to each other (as they have been measured against the same target).

S6 pChEMBL Range in Test Sets

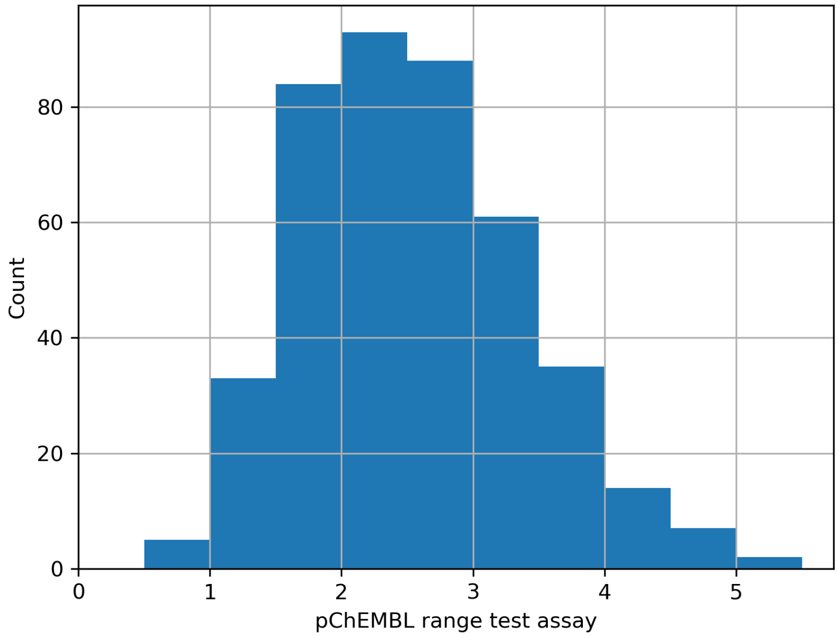

Figure S7: Distribution of the range (maximum - minimum) of pChEMBL values in the test sets.

We calculated the range (maximum - minimum) of pChEMBL values for each test set and plotted the distribution in Figure S7. The range of pChEMBL values was generally around 2.5 (95% CI of median: 2.3-2.6), but ranging from as small as 0.8 to as large as 5.2 log units. Considering the irreducible noise in bioactivity experiments (estimated to be around 0.3 [7]), the range of pChEMBL values of a test set has a great impact on the maximum performance that one can expect to achieve with a predictive model. In an assay with a small range, repeated experimental measurements could give different rankings of the measured compounds. This is important to keep in mind when evaluating predictive models.

S7 Model Consistency

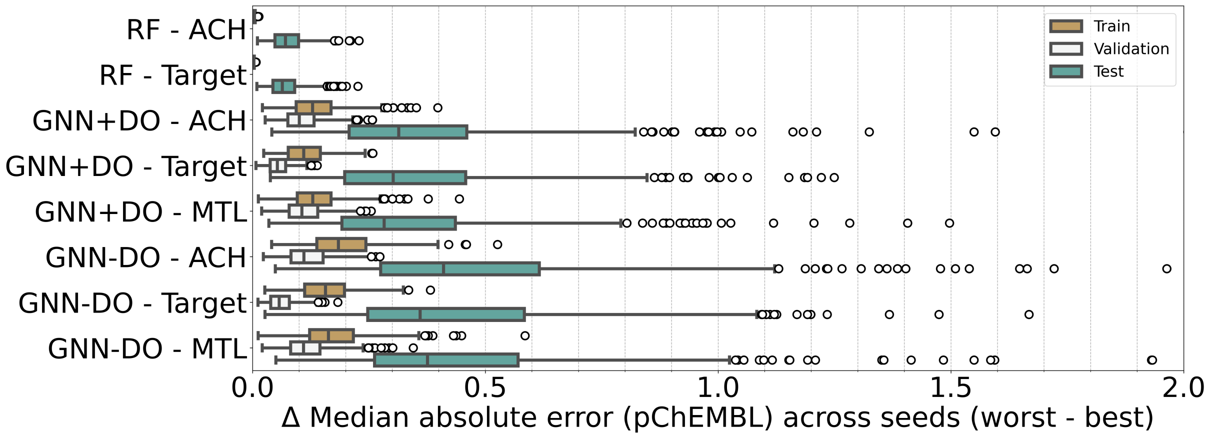

Figure S8: Boxplots of the distributions of variability across 422 test sets using ACH curation. Variability is quantified as the difference between the maximum and minimum median absolute error (medAE) in pChEMBL obtained across five random seeds. Results are shown for the training (orange), validation (white), and test (green) sets using RF regressors and GNNs trained with a dropout rate of 0.3 (GNN+DO) and without dropout (GNN-DO).

To evaluate the effect of the random seed on model performance, we evaluated all models with five different random seeds (Figure S8). The spread was defined as the difference in performance when comparing the best (lowest medAE) to the worst (highest medAE) result over the five seeds, keeping all other factors the same (i.e., same data and model set-up). For the GNNs, both set-ups without and with dropout (rate of 0.3) were evaluated on the test set. We observed very high variability in performance on the test sets with different random seeds using the GNNs, while the spread on predictive performance of the training and validation set was smaller (as expected). This is likely due to severe overfitting as the validation set in this experiment was designed to be very similar to the training set and the GNN models have many parametery relative to the training-set size. In contrast, RF regressor models showed generally low variability, even for the test set.

References

[1] Landrum, G. A.; Beckers, M.; Lanini, J.; Schneider, N.; Stiefl, N.; Riniker, S. SIMPD: An Algorithm for Generating Simulated Time Splits for Validating Machine Learning Approaches. *J. Cheminf.* **2023**, *15*, 119.

[2] Conover, W.; Iman, R. *Multiple-Comparisons Procedures. Informal Report*; 1979.

[3] Conover, W. J. *Practical Nonparametric Statistics*; John Wiley & Sons, 1999.

[4] Dunn, O. J. Multiple Comparisons Among Means. *J. Am. Stat. Assoc.* **1961**, *56*, 52–64.

[5] Demšar, J. Statistical Comparisons of Classifiers Over Multiple Data Sets. *J. Mach. Learn. Res.* **2006**, *7*, 1–30.

[6] Landrum, G. Thresholds for "Random" in Fingerprints the RDKit Supports. 2021; https://greglandrum.github.io/rdkit-blog/posts/2021-05-18-fingerprint-thresholds1.html#morgan-fps, RDKit blog.

[7] Brown, S. P.; Muchmore, S. W.; Hajduk, P. J. Healthy Skepticism: Assessing Realistic Model Performance. *Drug Discov. Today* **2009**, *14*, 420–427.
